# Reducing aggressive behavior in forensic inpatients with virtual reality aggression prevention training—intellectual disability: a pilot study

**DOI:** 10.3389/fpsyg.2026.1792178

**Published:** 2026-05-29

**Authors:** Patricia van Reekum, Frank van den Boogert, Peter de Looff, Wim Veling, Caro Jacobi, Lianne van de Griend, Stefan Bogaerts, Erik Masthoff

**Affiliations:** 1 Fivoor Academy of Research, Innovation & Development (FARID), Rotterdam, Netherlands; 2 Department of Developmental Psychology, School of Social and Behavioral Sciences, Tilburg University, Tilburg, Netherlands; 3 National Expertcentre Intellectual Disabilities and Severe Behavioral Problems, De Borg, Bilthoven, Netherlands; 4 Behavioural Science Institute, Radboud University, Nijmegen, Netherlands; 5 Universitair Medisch Centrum Groningen, Groningen, Netherlands

**Keywords:** aggression, arousal, Empatica E4, forensic, intellectual disability, virtual reality, VRAPT

## Abstract

**Background:**

Patients with mild to borderline intellectual disability (MBID) are overrepresented in forensic settings. In forensic psychiatric centers (FPCs), aggressive incidents are more frequent among offenders with MBID. Enhancing regular aggression regulation training with Virtual Reality (VR) technology could be effective by providing immersive roleplays and focusing on learning new behavior.

**Objective:**

This pilot study aimed to preliminary investigate the effects of the VR Aggression Prevention Training - Intellectual Disability (VRAPT-ID) on reducing aggressive behavior in forensic inpatients with MBID.

**Methods:**

A single case experimental design (SCED) study with a multiple baseline approach was conducted. Arousal levels during training sessions were monitored using the Empatica E4, a wristband that measured pulse rate (PR), pulse rate variability (PRV) and electrodermal activity (EDA). Aggressive behavior was measured using the Social Dysfunction and Aggression Scale (SDAS). Therapeutic alliance was assessed with the Session Rating Scale (SRS), a qualitative questionnaire.

**Results:**

Five out of 10 participants completed the entire training. The results showed that VRAPT-ID was associated with a decrease in aggressive behavior in four of the five participants who completed the intervention. Analysis showed that skin conductance responses (electrodermal activity), as an indicator of arousal, increased significantly during training sessions, which may reflect increased physiological activation during sessions, although no direct link with behavioral change was established. No significant changes were observed in pulse rate (PR). Pulse rate variability (PRV) data contained too many artifacts and therefore could not be reliably analyzed. Regarding therapeutic alliance, all participants who completed the training reported that VRAPT-ID helped them manage anger more effectively and that they found the training enjoyable.

**Conclusion:**

The results of this pilot study suggest that VRAPT-ID may be a feasible and potentially promising intervention for addressing aggressive behavior in individuals with MBID in a forensic setting. Future research, including RCT, is needed to further examine these preliminary findings and to investigate the proposed underlying mechanisms of VRAPT-ID.

## 1 Introduction

Patients with mild to borderline intellectual disabilities (MBID; IQ 50–85) are overrepresented in forensic settings (Mosk and Degraeve, 2019). MBID is characterized by deficits in intellectual and adaptive functioning (American Psychiatric Association, 2013) and increases the vulnerability to criminal behavior (Janković et al., 2021). The MBID population exhibits considerable heterogeneity, with marked differences observed in cognitive abilities, individual traits, comorbid psychiatric conditions, environmental backgrounds, and responsiveness to stress (Douma et al., 2007; Nouwens et al., 2017). Forensic patients with MBID often exhibit increased impulsivity and other risk factors for aggressive and violent behavior (Garritsen et al., 2024).

Aggressive behavior is common in forensic psychiatric settings (Klein Tuente et al., 2021; Neumann and Klatt, 2021; Schuringa et al., 2019). Based on staff monitoring, Klein Tuente et al. (2021) showed that 86% of 120 Dutch inpatients displayed moderate aggressive (defined as short outbursts of aggressive talking; or repeated episodes of throwing trivial things, hitting objects, or slamming doors), 65% of inpatients displayed severe aggressive behavior (for example: ostentatious in opposing the rules of social interaction; being completely uncooperative; dangerously assaulting a member of staff; making serious insults and/or wishing people harm), and 37.5% of inpatients showed physical aggression.

This aggressive behavior is more frequent among offenders with MBID, leading to negative consequences for victims and disrupting the patient rehabilitation (Chester et al., 2018). Aggressive incidents also negatively affect the therapeutic climate in forensic settings (Neumann and Klatt, 2021). Traditional psychological treatments, particularly those based on cognitive behavioral therapy (CBT), often prove ineffective for patients with MBID (Henwood et al., 2015; Janković et al., 2021). These patients struggle to process verbal information and generalize learned skills, making it difficult to apply acquired knowledge in real-life situations (Kleinert et al., 2009). Patients with MBID require personalized tailored therapeutic interventions that consider their specific cognitive abilities and limitations (Janković et al., 2021; Kip et al., 2018). A recent systematic review by Prior et al. (2023) found moderate evidence supporting the efficacy of CBT for individuals with ID, but only when adapted to their specific needs. This highlights the importance of specialized and personalized treatment strategies that align with the patients’ cognitive capacities to enhance treatment outcomes and improve the generalization of learned skills.

In addition to personalization and generalization, VR may enhance therapeutic alliance. This is crucial in forensic psychiatry, where therapy resistance and low compliance are common, further limiting the effectiveness of psychological interventions (Brunner et al., 2019; Dixon et al., 2016; Kip et al., 2018; Main and Gudjonsson, 2006). Therapeutic alliance is based on the principles of Bordin (1979) and compromises agreement on (1) tasks, (2) goal alignment, and (3) the development of bond. VR-based training may strengthen these components in forensic psychiatric populations through several mechanisms. VR offers immersive, interactive simulations that closely resemble real-world situations, thereby enabling individuals to engage more directly with tasks (Riches and Kaleva, 2025). Embedding concrete therapeutic tasks in immersive simulations, reduces abstraction and enhances transparency plus focuses on learning new behavioral skills through VR experience rather than relying on verbal information processing.

Embedding goal alignment can directly mirror real-life risk situations such as social conflicts on the ward and setting boundaries. This ecological validity allows therapist and patient to collaboratively identify personally meaningful behavioral goals within simulated environments. Lastly, embedding bond development may reduce interpersonal threat. The futuristic look of the VR equipment and immersive role-plays boost therapy compliance, particularly in hard-to-engage forensic patients (Kip et al., 2019; Klein Tuente et al., 2018).

In conclusion, the interactive VR approach enables patients to observe, experiment with different responses, and learn from their outcomes, thereby enhancing interpersonal effectiveness within a safe and controlled environment (Howard and Gutworth, 2020), which may ultimately contribute to strengthening the therapeutic alliance.

The use of VR in therapy aligns with the Risk-Need-Responsivity (RNR) model (Bonta and Andrews, 2007). Regarding the Need principle, aggression regulation is conceptualized as a dynamic criminogenic need in forensic psychiatry. A major issue in forensic psychiatry is the lack of exposure to real-world situations where management skills can be practiced (Ivarsson et al., 2023). Virtual Reality (VR) offers the possibility to practice new behaviors in a simulated, safe environment within aggression regulation therapy (Benbouriche et al., 2014; Freeman et al., 2017; Pan and Hamilton, 2018). VR can therefore directly address the need of aggression regulation allowing patients to practice adaptive responses within simulated high-risk interpersonal situations. The Responsivity principle is especially important for the heterogeneous population of individuals with MBID whose treatments should be adapted to their cognitive abilities (Didden et al., 2019; Janković et al., 2021), for example through experiential and visually supported learning, ultimately aiming to reduce recidivism (Polaschek, 2012). The use of VR also aligns with the responsivity principle, as immersive environments can facilitate experiential learning and visually supported behavioral rehearsal. In addition, VR allows graded exposure to emotionally salient interpersonal situations and may support the recognition and differentiation of a broader range of affective states beyond anger (e.g., fear, shame, frustration and rejection), thereby supporting emotion regulation and risk management. A review by Sygel and Wallinius (2021) of current VR treatment methods highlighted the lack of large clinical studies in forensic psychiatry, although several VR interventions were deemed feasible and acceptable.

Klein Tuente et al. (2018) developed Virtual Reality Aggression Prevention Training (VRAPT) to improve aggression regulation in social situations. They conducted a randomized controlled trial (RCT) with 128 forensic psychiatric patients from four Dutch FPCs. No significant improvements were observed in the VRAPT condition compared to a waiting list group. Thirteen patients dropped out for various reasons, such as drug use, security restrictions and transfer to another clinic. Klein Tuente et al. (2020) identified several limitations that might account for these findings. For instance, the authors indicated that self-report measures were too difficult for some patients which might have resulted in null findings. Another limitation was that the most severe aggressive patients were excluded in the study, which resulted in fewer opportunities to reduce aggressive behavior. In addition, the VR intervention appeared overly complex and contained a substantial amount of theory, despite the exclusion of patients with MBID.

More recently, several studies on slightly altered VRAPT derivatives have been published. The feasibility study of Woicik et al. (2023) reported positive effects on aggression, anger, and emotion regulation, in a prison-based population. The pilot study of Ivarsson et al. (2023) showed probable changes in criminogenic needs related to recidivism risk in incarcerated violent offenders. Previous studies on VR aggression training in forensic patients excluded participants with MBID (IQ < 70) (Ivarsson et al., 2023; Klein Tuente et al., 2018; Woicik et al., 2023). However, these patients are believed to benefit substantially from this intervention. VR is experiential learning and places less demand on cognitive and verbal skills, which makes this a suitable form of therapy. VRAPT-ID was developed as an adaptation of the VRAPT protocol, specifically for adolescents and adults with MBID and aggression problems. Geraets et al. (2023) implemented VRAPT-ID in a non-forensic setting for aggression training. and reported that VRAPT-ID might be valuable for improving self-regulation of aggression, in which participants recognize triggers and apply learned strategies to prevent escalation in social situations (Geraets et al., 2023). The intervention was well-accepted by individuals with MBID and their relatives, mentors, and therapists (Geraets et al., 2023). However, their study lacked instruments for measuring aggression due to implementation problems, hence no valid information on changes in aggression could be provided (Geraets et al., 2023). The VRAPT-ID protocol in the current study is an adapted version based on the study by Geraets et al. (2023), Ivarsson et al. (2023), Klein Tuente et al. (2018), and Woicik et al. (2023), released in 2021.

One of the primary frameworks for understanding the origin and expression of aggression is the General Aggression Model (GAM; Allen et al., 2018), which serves as theoretical foundation of understanding and conceptualizing aggression with VRAPT-ID. The GAM is a comprehensive and integrative model that explains human aggression by incorporating social, cognitive, developmental, and biological factors. In accordance with this model, VRAPT-ID uses provocation and threats during VR sessions to trigger aggressive behavior. In combination with person factors like MBID, these situational factors influence cognition, affect, and arousal. When an individual encounters a threat, the brain signals a stress response, causing arousal (Roozendaal et al., 2009). In the final phase of GAM, the individual appraises the situation and decides how to respond. This action influences both the encounter and future responses, creating a feedback cycle (Allen et al., 2018).

Regarding the mentioned arousal when facing a threat, Bandura’s (1978) learning theory suggests that physiological stress stimulates alertness and focused attention, enhancing memory (Joëls et al., 2006; Squire et al., 2015). Besides, stress hormones enhance long-term memory storage (Roozendaal et al., 2009). However, it is important to note that this relation is not linear and there exists a zone of optimal autonomic functioning that enhances an individual’s learning capacities, known as the Yerkes-Dodson law of optimal arousal (Broadhurst, 1957; Yerkes and Dodson, 1908). This law posits that moderate levels of arousal facilitate performance, whereas excessive arousal impairs prefrontal regulatory functioning and executive control. The “optimal arousal level” thus promotes effective learning transfer and optimizes performance across cognitive, affective, and behavioral regulatory domains (Faller et al., 2019; Khazaei et al., 2021; Rozenek et al., 2019).

In VRAPT-ID, it is expected that arousal levels increase during the VR session after exposure to a trigger, promoting focused attention and consolidation that are required for learning and sequential reproduction (Allen et al., 2018). However, although heightened arousal can enhance learning performance, it is crucial that arousal levels do not become excessively elevated during VR sessions. VRAPT-ID is therefore expected to induce a moderate level of arousal to facilitate entry into the “optimal regulatory window”. Besides, VRAPT-ID combines graded exposure to emotionally salient interpersonal triggers with structured skills training, each of which involves distinct learning mechanisms and carries different risks. Arousal levels will be continuously monitored during the sessions, and appropriate measures will be implemented if signs of excessive arousal are observed. This is particularly relevant given the substantial heterogeneity within the MBID population, which may influence optimal arousal thresholds and responsivity to VR-based exposure. Physiological arousal levels can be assessed through physiological indicators reflecting the contribution of the sympathetic and parasympathetic branches of the autonomic nervous system. The sympathetic branch is primarily associated with mobilization and popularly associated with the fight-or-flight response, facilitating for example physiological readiness and heightened alertness (Everly and Lating, 2019). The parasympathetic branch facilitates restorative processes, popularly associated with the rest-and-digest response (Kamath et al., 2016; Trimmel, 2015). Electrodermal activity (EDA) provides a relative direct index of sympathetic arousal. Parasympathetic modulation can be estimated with some heart rate variability (HRV) indices, particularly those reflecting vagal activity (Jarczok et al., 2013; see Table 1 for an overview). Pulse rate (PR) reflects the net balance of sympathetic and parasympathetic influences on cardiac activity. Pulse rate variability (PRV) can approximate HRV but is influenced by vascular and measurement elements. It is anticipated that immersive VR will lead to increased arousal within a session, reflected by increased EDA, reduced PRV and increased PR.

**Table 1:** Baseline characteristics of the sample (*N* = 10).

	M (SD) or *N* (%)			Completers *N* = 5
	Non completers *N* = 5	Total *N* = 10
Age	40.80 (9.149)	36.20 (4.207)	38.5 (7.138)
Number of TBS treatment attempts	1.4 (0.548)	1.8 (0.837)	1.6 (0.699)
Schizophrenia	1	1	2
Unspecified psychotic disorder	1	2	3
Bipolar disorder	1	0	1
Neurocognitive disorder	0	0	1
Antisocial personality disorder	3	4	7
Borderline personality disorder	1	0	1
Paranoid personality disorder	1	0	1
Narcistic personality disorder	1	0	1
Otherwise specified personality disorder	2	2	4
Autism spectrum disorder	1	0	1
ADHD	0	1	1
Alcohol abuse	3	3	6
Cocaine abuse	3	3	6
Cannabis abuse	4	3	7
Amphetamine abuse	2	3	5
Gambling abuse	1	1	2
Other substance disorder	1	2	3

This pilot study is the first to examine the effectiveness of VRAPT-ID in reducing aggressive behavior in forensic inpatients with MBID. Our first hypothesis is that VRAPT-ID leads to a reduction of aggressive behavior over time within individual participants. Additionally, we examined the effect of the immersive VR on the levels of physiological arousal. The second hypothesis posits that the immersive VR features of VRAPT-ID, combined with exposure to socially challenging scenarios, will elicit a moderate level of arousal and to facilitate entry into the “optimal regulatory window” during VR training. The third hypothesis proposes that arousal levels will be elevated during sessions 7 to 12 relative to sessions 1 to 6, reflecting the increased use of personalized and socially provocative role-plays introduced in the second phase of the training. Lastly, this study will also focus on therapeutic alliance as this is an important factor to enhance effectiveness. Our fourth hypothesis is that VRAPT-ID enhances therapeutic alliance in this forensic population.

## 2 Materials and methods

### 2.1 Study design

The present study utilizes a single case experimental design (SCED). Details of this design are reported following the SCRIBE guidelines (Single Case Reporting Guideline in Behavioral Interventions) (Tate et al., 2017). The aim of single case studies is often not to evaluate whether the results of an intervention can be generalized to the population, but to assess whether an intervention is effective in a specific subgroup (Bouwmeester and Jongerling, 2020). Specifically, this study used a non-concurrent multiple baseline design (MBD), because no ‘washout’ effect or immediate effect of the intervention was expected. The study procedure consisted of three phases: *baseline phase (A) – experimental phase (B) – follow-up phase*. All participants entered the baseline phase of study at the same time, after which the intervention phase was sequentially introduced. This MBD can be seen as multiple AB designs, with as many AB designs as there are participants (Krasny-Pacini and Evans, 2018).

### 2.2 Procedure

This study was conducted between October 2022 and August 2023. Ten participants were randomly assigned to one of five different baseline lengths, ranging from 5 to 9 weeks. All participants entered baseline phase A at the same time, during which the primary outcome variable, observed aggressive behavior, was measured. Every week, two participants transitioned into the intervention phase, with randomization determining the starting moments. The sequential introduction helped to visualize the lack of retest effect and unrelated progress in participants who had not yet undergone the intervention (Krasny-Pacini and Evans, 2018). Randomization was conducted using a web-based scientific randomization program^1^, based on previously described methods (Bouwmeester and Jongerling, 2020; Bulté and Onghena, 2009; Wampold and Worsham, 1986). During the experimental phase B, participants received the VRAPT-ID intervention, consisting of 12 weekly sessions. The primary outcome variable was measured continuously weekly, and additional outcomes (i.e., arousal and therapeutic alliance) were measured during training sessions. The intervention phase duration could vary due to circumstances such as illness, holidays of therapists, other clinical activities or no-shows. The final follow-up phase started immediately after the experimental phase B and lasted for 12 weeks, during which only observed aggressive behavior was measured. The total study duration was at least 29 weeks, depending on the lengths of the baseline and intervention phases: a minimum of 5 weeks baseline + a minimum of 12 weeks intervention + 12 weeks follow-up.

### 2.3 Sample size

In a multiple baseline design with follow-up according to the What Works Clearinghouse criteria (What Works Clearinghouse, 2020), at least three participants and a minimum of five data points per phase are required (Krasny-Pacini and Evans, 2018; Kratochwill et al., 2023). The number of possible start moments for the intervention significantly affects the study’s power (Bouwmeester and Jongerling, 2020). In general, more measurements yield a more reliable test statistic due to the larger number of observations (Bouwmeester and Jongerling, 2020). Given these criteria and to account for possible dropouts, the required sample size was set at 10.

### 2.4 Setting

This study was conducted at *FPC De Kijvelanden*, part of Fivoor, one of seven forensic psychiatric centers in the Netherlands. Under Dutch law, an FPC admits psychiatric patients with a TBS order (terbeschikkingstelling: literally translated ‘at the discretion of the state’). This provision allows for treatment, possibly following a partially served prison sentence, for mentally disordered offenders (Van Marle, 2002). Patients residing in an FPC have committed serious violent crimes, have been assessed by the court as reduced to not accountable due to the impact of their psychopathology on their offenses, and have an estimated high risk of reoffending. FPC de Kijvelanden specializes in treating patients with MBID and has three specific wards for these patients. The study protocol was approved by the Medical Ethical Committee Brabant (METC number: NL81300.028.22). There was no funding provided for this study.

### 2.5 Participants

All 10 participants were male, with an average age of 38.5 (*SD* = 7.138) ranging from 27 to 49 years. Inclusion criteria required patients to have aggression regulation problems as indicated by their head of treatment, and a diagnosis of MBID according to codes 317 or 318 of the Diagnostic and Statistical Manual of Mental Disorders-5 (American Psychiatric Association, 2013). The diagnosis of MBID was established by a qualified psychologist through assessments of cognitive and adaptive skills following guidelines from the National Knowledge Centre for Intellectual Disabilities (Landelijk Kenniscentrum LVB, 2024). Cognitive functioning was measured using the Wechsler Adult Intelligence Scale, Fourth Edition (WAIS-IV; Wechsler, 2008) and adaptive skills with the Adaptive Ability Performance Test (ADAPT; Jonker et al., 2021). These assessments were not administered specifically for this research but were part of the standard diagnostic process. Exclusion criteria included experiencing an active psychotic episode or receiving other aggression regulation therapies simultaneously. Participants did not receive compensation for their participation.

Heads of treatment and other staff members, such as social workers, were informed about the study through presentations, staff meetings, and/or e-mails. Heads of treatment were asked to preselect patients who met the inclusion criteria. Preselected patients were first informed about the study by the researcher and given an information leaflet. To ensure full understanding considering their MBID, the information was also provided to each patient’s coach (social worker from the ward). After 1 week, participants were asked if they were willing to participate. Written informed consent was obtained from those who agreed.

**Figure 1:**
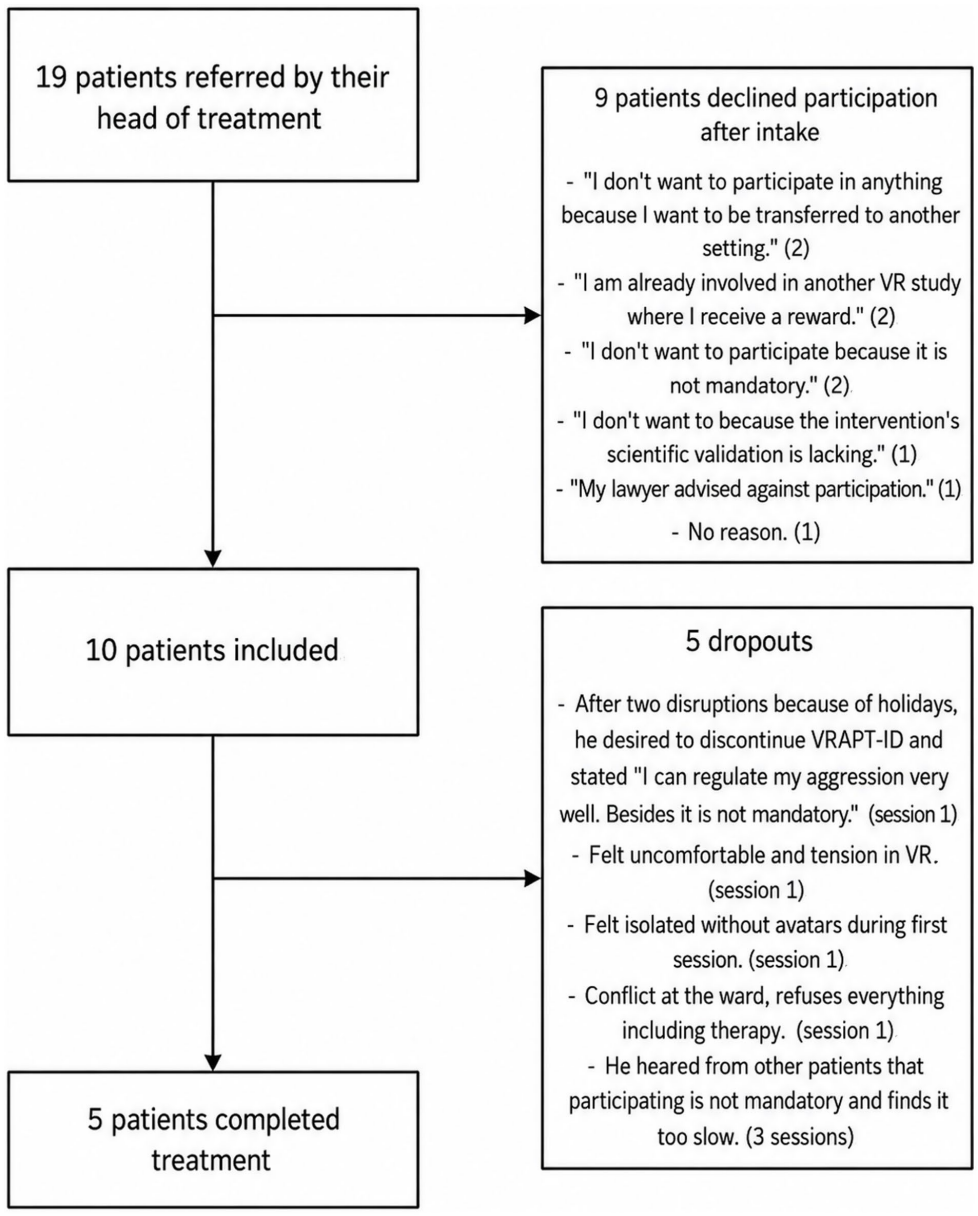
Flow chart of the pilot.

In total, 19 participants were initially preselected by the heads of treatment for this study. After the informed consent process, 9 patients declined for various reasons. For example, at the same time another study was being conducted at the FPC in which VR was used as an assessment tool. Enrolling in both studies was allowed, but for the other study patients did receive a reward. Detailed information about the inclusion process, including reasons for drop-out, is presented in Figure 1. Half of the participants had undergone one or more previous TBS treatment attempts in another clinic, with an average of 1.6 attempts (*SD* = 0.699), ranging from 1 to 3 attempts. Four participants dropped out after one session and one participant after three sessions. This left five participants who completed the entire intervention. Since this was a single case experimental design, only the participants who completed the intervention were analyzed to examine changes in aggressive behavior over time. The baseline characteristics of the total sample are shown in Table 1. Given the small number of participants in both groups (five completers and five non-completers), no statistical comparisons were conducted, and no conclusions can be drawn regarding potential differences between completers and non-completers.

### 2.6 Intervention

The main goal of VRAPT-ID is for patients to learn and practice new behaviors, to re-evaluate social situations, and teach skills to cope with patients’ anger more appropriately. In addition, VRAPT-ID involves social workers from the ward, who serve as patients’ coaches, as part of the social network to learn what can help patients to deal with their anger and help in transferring skills learned during VR into daily life.

VRAPT-ID is an adaptation of the original VRAPT (Klein Tuente et al., 2018) to better suit the MBID patient group (Geraets et al., 2023). The feasibility study of Geraets et al. (2023) suggested increasing VR practice time, involving the social network more, and personalizing the number and frequency of sessions. The original Social Information Processing (SIP) model (Crick and Dodge, 1994) used in VRAPT was replaced by the simpler ‘Stop - Act - Think - Do’ (SODA) principle. Patients learned to stop or exit unpleasant or stressful situations (S = “stop”), create a short moment of relaxation (*R* = “relax”), think about how to respond (*T* = “think”) and then act (A = “act”). This ‘Stop- Relax-Think-Act’ strategy was a key focus and was repeatedly practiced in VR environments. Additionally, the language of the training materials was modified for the MBID target group, and relaxation exercises were added to the end of each session.

During the VRAPT-ID intervention, patients practiced different interactive virtual role-plays. The scripts and role-plays were written by both the patients and trainers. Unlike real-life situations, patients could click ‘pause’ and remove the VR glasses as a quick and safe escape from the virtual environment. VRAPT-ID allowed the trainer to compliment patients and prompt them to use ‘Stop - Act - Think - Do’ skills with a headset. This dynamic and interactive nature of the VR system meant VRAPT-ID could be tailored to the specific needs of the patients, allowing them to practice their own learning goals and difficulties. Twice, the coach of the patient (a social worker from the ward) joined a VR session to provide input on the role-plays regarding triggers that cause aggression in patients on the ward. Conversely, during the session the social worker gained insight into how aggression develops in the patient and which strategies might be beneficial, such as the SODA principles. VRAPT-ID consisted of 12 individual training sessions. Regarding arousal, VRAPT-ID aims to achieve the “optimal regulatory window”. Arousal was monitored by trainers through behavioral observation and with the use of the Empatica E4 device. Excessive arousal, for example loss of engagement with the scenario or visible signs of distress, was managed by pausing the scenarios, addressing the patient directly using the trainer’s own voice instead of the avatar’s voice, and modulating the intensity of the social interaction. In line with that, an important component of the VRAPT-ID intervention is the use of VRelax (Veling et al., 2021) at the end of each session. This element is intended to reduce arousal when tension levels have become high. Findings from a recent meta-analysis (Kjærvik and Bushman, 2024) indicated that arousal-decreasing activities effectively reduce anger and aggression as opposed to arousal-increasing activities. Moreover, their results suggest that such activities benefit criminal offenders and non-offenders equally, and that individuals with intellectual disabilities derive similar benefits as those without such disabilities. Jones et al. (2008) posit that this may be explained by the universality of physiological arousal responses, which are not directly associated with cognitive ability. Assessing treatment integrity is challenging in this context. Treatment integrity was supported using a standardized treatment protocol and regular intervision of therapists. However, the role-plays varied across participants because they were tailored to individual situations and needs. This personalization is an intentional component of the intervention and is clinically valuable; however, it may have implications for treatment integrity.

Each VRAPT-ID session has the same structured format:

- Complete the *Outcome Rating Scale* (ORS; Hafkenscheid et al., 2010; Miller et al., 2003): Patients rated how they felt on a scale from 0 to 10. This standard therapeutic assessment was not used in this study as it did not relate to our research questions.
- Complete the ‘stress meter’ (in Dutch: boosheidsmeter): This helped patients gauge their current level of stress or anger.
- Learn about emotions and sensations: Patients learned to identify emotions, facial expressions, bodily sensations, and how these sensations manifest in provocative situations.
- Role-plays: These were based on recent personal experiences of the patient, allowing them to practice in realistic situations.
- Learn and practice ‘Stop - Act - Think - Do’ skills: Each session included practicing one or more of these skills to manage their reactions.
- Complete the *Session Rating Scale* (SRS; Duncan et al., 2003): Patients rated the sessions on a scale from 0 to 10 to provide feedback on their experiences.
- Calm-down exercise: Each session concluded with an exercise to help patients relax, especially after the often-increased tension during the session. This included using the ‘VRelax’ (Veling et al., 2021). The VRelax is a head-mounted display with 360 degrees videos. Patients are immersed in calming and immersive virtual environments which help shift attention away from negative emotions and promote relaxation. VRelax has been scientifically and CE-MDR certified.

#### 2.6.1 Trainers

All psychologists, expressive therapists, and general remedial educationalists (in Dutch: orthopedagoog-generalist) were invited to participate as VR trainers for this study. Those who volunteered received training in using VR within the therapeutic context of the VRAPT-ID intervention by certificated trainers to ensure sufficient skill and proficiency. Seven psychologists, one creative therapist, and one general remedial educationalist were trained. During the intervention weeks, several intervision meetings were held led by one of the researchers, where trainers could share progress, ask questions, and learn from each other, ensuring the intervention protocols were followed. Social workers from the ward, who served as patient coaches, were involved in two sessions of the intervention. Because the VR equipment and the Empatica E4 wristband were new to the trainers, each VR session was conducted by two trainers.

#### 2.6.2 Social worlds VR-cognitive behavioral therapy (CBT) software

**Figure 2:**
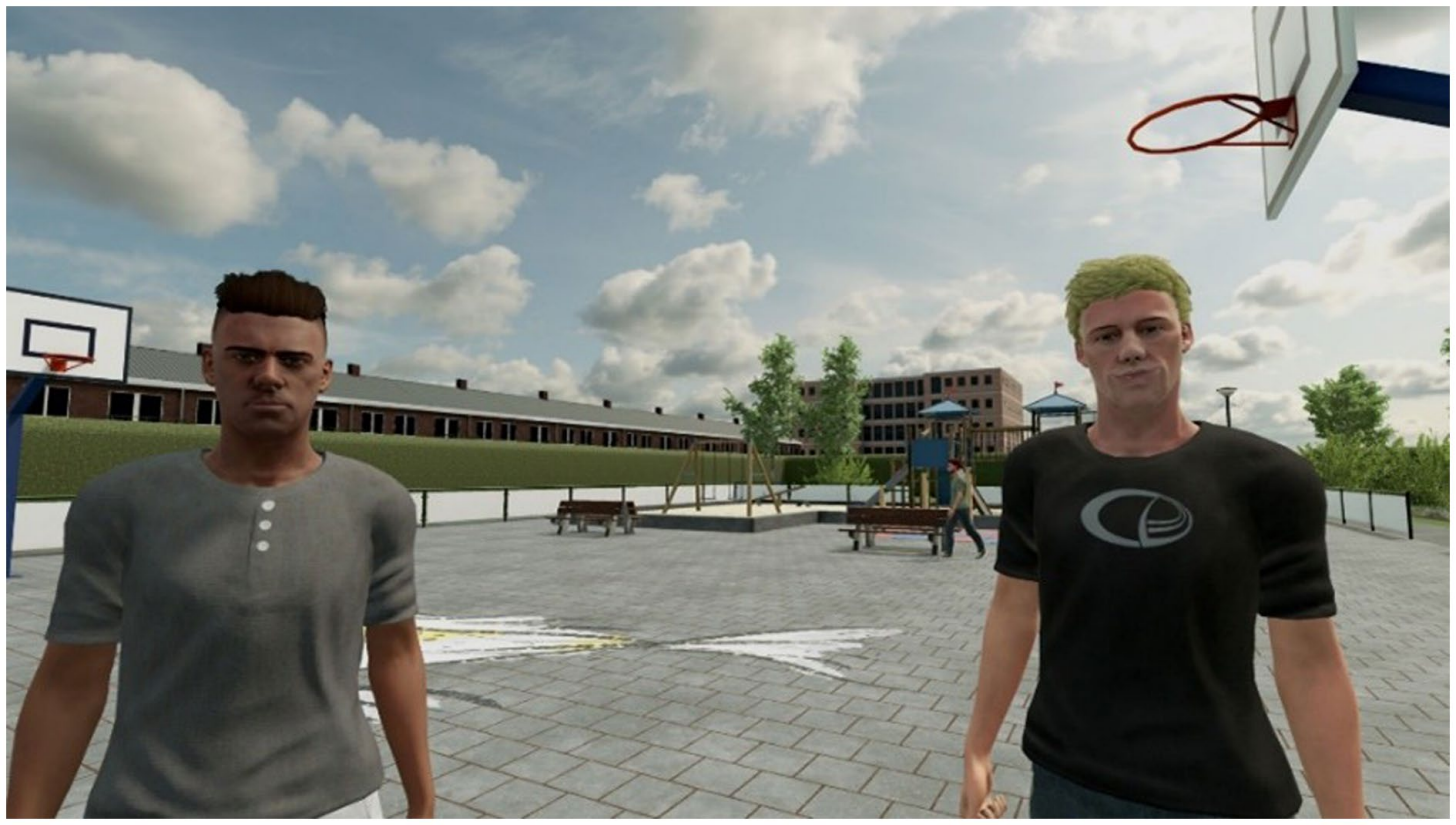
Impression of ‘Social Worlds 4.1’. Reproduced with permission from CleVR BV (2023).

The *Social Worlds* VR-CBT software for this intervention was provided by CleVR BV, a company specialized in producing VR applications for people with psychiatric disorders. CleVR’s Social Worlds VR-CBT software received the CE mark as a medical device. Social Worlds VR-CBT was also used in several VRAPT studies (Klein Tuente et al., 2018; Woicik et al., 2023) and provides a three-dimensional virtual environment where participants can practice social situations and interactions. This study employed the renewed ‘Social Worlds 4.1’ software, which includes more environments and new possibilities. An example of the VR world in ‘Social Worlds 4.1’ is shown in Figure 2.

#### 2.6.3 Hardware

**Figure 3:**
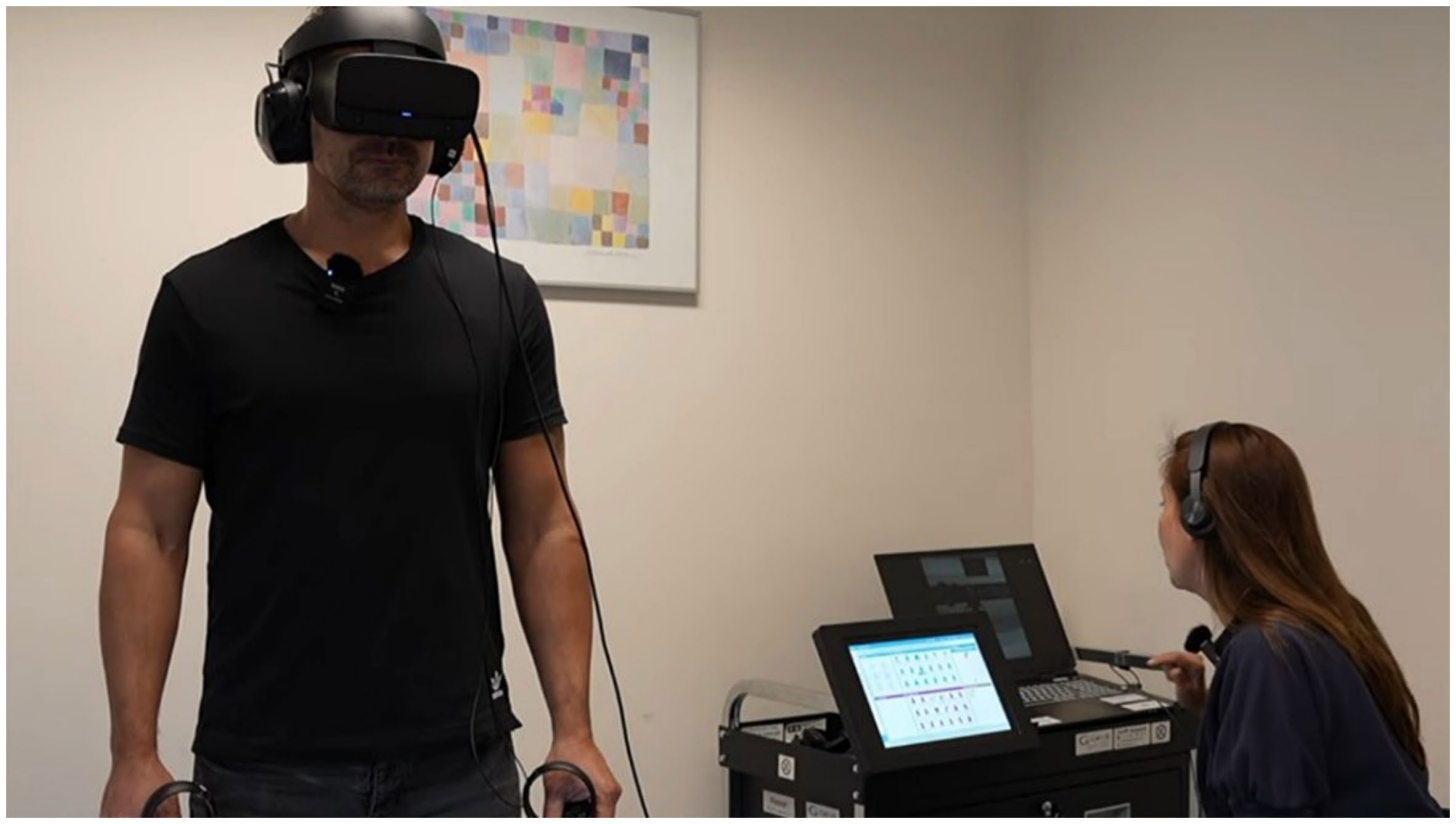
The VR setup. Reproduced with permission from people on the picture.

Participants wore a head-mounted display (Oculus Rift S 2) and noise-cancelling headphones, and they navigated using two Microsoft Xbox One Controllers. Trainers communicated with participants through headphones equipped with a microphone and controlled the virtual environment using a tablet. Participants interacted with a virtual character (avatar) operated and controlled by the trainer. The trainer, assuming the role of the virtual character, used a microphone with voice distortion for speech and manually controlled the facial expressions and body movements of the virtual character. The trainer always had full control over the virtual environment and could immediately change and/or stop the virtual environment if necessary. An example of the VR setup is shown in Figure 3.

The ‘VRelax’ was used after every session for a relaxation exercise (Veling et al., 2021). VRelax is an Oculus VR head-mounted display featuring an easy-to-use VR self-management relaxation tool with immersive 360° nature videos and interactive animated elements.

### 2.7 Measurements

No extensive self-report questionnaires were included in the study procedure because patients with MBID often struggle with reading long sentences and understanding verbal information, which can make their answers unreliable or invalid (Willner et al., 2019). Therefore, a combination of short self-report questionnaires, staff-rated observation scales, and biological measures was used. Sociodemographic characteristics such as age, number of TBS treatment attempts, and psychiatric disorders were collected and are shown in Table 1.

#### 2.7.1 Self-report

The *Session Rating Scale* (SRS; Duncan et al., 2003) was used to measure therapeutic alliance. The SRS is a four-item visual analogue instrument designed to help participants identify any issues with the therapist or therapy. Originally developed by Johnson (1995) as a 10-point Likert scale, the SRS Version 3 was later shortened to four visual analogue items, making it less time-consuming (Duncan et al., 2003). The reliability of the SRS is supported by Cronbach’s alpha coefficient of 0.89. The SRS was self-reported after each VRAPT-ID session to evaluate their session experiences. This information was then used to determine which elements of the pilot training enhanced therapeutic alliance and which elements decreased it.

One short qualitative questionnaire was administrated once at the end of the VR intervention to measure therapeutic alliance. This questionnaire consisted of five questions designed by the researcher to gather participants’ opinions on VRAPT-ID and VR training in general. The questions were brief to ensure they were easily understood by the target group. For example: How do you feel about participating in this VR training? What did you like/ dislike? Additionally, trainers completed a separate questionnaire with open-ended questions to provide their feedback on VRAPT-ID. For example: How do you think this training helped the patient with his anger? Which exercises were helpful? Which were not?

#### 2.7.2 Psychophysiological measures

The *Empatica E4* (E4; Garbarino et al., 2014; Schuurmans et al., 2020) was used to measure several arousal related measures during the sessions. The E4 is a wristband that measures electrodermal activity (EDA), blood volume pulse (providing inter beat interval and pulse rate), body temperature, and accelerometry (de Looff et al., 2019).

In the current study Pulse Rate (PR), Pulse Rate Variability (PRV), and Electrodermal Activity (EDA) were used as indicators of autonomic arousal. PR, derived from photoplethysmography, was expressed in beats per minute. PRV was quantified using the root mean square of successive differences (RMSSD), expressed in milliseconds (ms). RMSSD reflects short-term variability in inter-beat-intervals and is often interpreted as an index of vagally mediated cardiac modulation. PRV measured with PPG represents an approximation of HRV as it is also influenced by vascular and measurement factors. EDA reflects changes in the skin’s electrical conductance associated with sympathetic nervous system activation (Boucsein, 2012). The signal consists of a tonic component, the Skin Conductance Level (SCL), and phasic Skin Conductance Responses (SCR) which represent increases in response to specific stimuli or spontaneous fluctuations (Dawson et al., 2007). EDA is expressed in microsiemens (μS). Physiological data were recorded continuously and preprocessed with the wearables package for artifact detection and feature computation (de Looff et al., 2022).

#### 2.7.3 Staff-rated measures

Aggressive behavior in participants was measured with the *Social Dysfunction and Aggression Scale* (SDAS-9; Kobes et al., 2012; Wistedt et al., 1990). For each participant, this questionnaire was completed by social workers from the ward on a weekly basis. Scoring was conducted either via Qualtrics or on paper. The SDAS systematically records staff observations on a wide range of aggressive behaviors. The instrument consists of 11 items, each scored 0 to 4, where 0 = ‘not present’ and 4 = ‘very serious’. Higher scores indicate higher levels of aggressive behavior. With a maximum possible score of 44. An example of an item is: “Physical aggression towards staff (e.g., kicking and hitting)”. The Dutch version of the SDAS demonstrates moderate inter-rater reliability (ICC = 0.50; Kobes et al., 2012) and good convergent validity in an FPC (Cronbach’s alpha (11 items) = 0.82; Kobes et al., 2012).

### 2.8 Statistical analysis

Data was analyzed using R and SPSS. Prior to the main data analysis, all data were inspected for deviations such as missing values and outliers. Descriptive statistics were used to examine the potential presence of abnormal response patterns. First, visual analyses were performed using the application of Bouwmeester (2021) to determine whether there was an association between the VRAPT-ID intervention and the primary outcome measure, aggressive behavior. Data on these dependent variables were presented in time series graphs under different experimental conditions (baseline phase A, intervention phase B, and follow-up phase) for individual participants. The visual analysis involved within-phase data examination and making between-phase comparisons (Lobo et al., 2017). Within-phase data examination comprised evaluating the level, trend, and stability of the data within each phase. The level was estimated using the mean and median, whereas the trend was evaluated by determining whether the data points were monotonically increasing. Within-phase stability was assessed by calculating the percentage of data points within 15% of the phase median. The stability criterion was satisfied if about 85% of the data in a phase fell within a 15% range of the median of all data points for the phase (Lobo et al., 2017). For between-phase comparisons, immediacy of effect, consistency of data patterns, and overlap of data between baseline and intervention phases were examined. Non-overlap of All Pairs (NAP) is a non-parametric method used to measure behavior change in single-case research. It is an index of data overlap between phases (Parker and Vannest, 2009). NAP has some limitations, such as a ceiling effect and a tendency to overestimate the effect size when there is a trend. Therefore, a Tau-U approach was also used. Tau-U is another metric used in SCEDs to measure the effectiveness of the intervention. Tau-U combines nonoverlap between phases with intervention phase trend and can correct for an undesired baseline trend (Lee and Cherney, 2018; Parker and Vannest, 2009). However, both NAP and Tau-U have a limitation known as the problem of typicality. This means they do not account for the varying probabilities of nonoverlap between cases due to different baseline scores (Landman et al., 2024). For instance, it is statistically more difficult for a participant with a high baseline score to improve compared to one with a low baseline score. To address this issue, the Typicality of Level Change (TLC) index was additionally conducted (Landman et al., 2024). TLC is a third metric used in SCED research to assess whether changes during the intervention phase are typical or extraordinary compared to baseline data. TLC is based on the logic of typicality tests (Rouanet et al., 2000) and shows the probability of observed improvement compared to any possible improvement, conditioned on the baseline score. If visual analyses revealed at least three demonstrations of an intervention effect, indicating a functional relationship between the VRAPT-ID intervention and aggressive behavior, quantitative analyses were performed to evaluate the magnitude of the intervention effect. Effect sizes were calculated for each participant using standardized mean difference *Hedges’ g,* an extension of *Cohen’s d* recommended for single case experimental studies as it corrects for small sample sizes (Lobo et al., 2017). A randomization test (Bouwmeester, 2021) was also performed to measure the chance of observing the exact pattern found, given all possible data patterns.

Regarding the SRS data, NAP was calculated to test whether therapeutic alliance increased as the training progressed. To do this, the SRS was standardized. The SRS was measured on a 92 mm line, and patients’ answers were standardized to a 92-point scale. Since NAP can only compare two phases, the SRS data was split into two periods. T1 (sessions 1 to 5) and T2 (session 6 to 12). This division was chosen because, from session 6 onwards, patients practiced with their own experiences.

For the physiological recordings with the Empatica E4, we examined whether arousal increases throughout the session, as operationalized by decreasing PRV, increasing PR, increasing SCL and increasing peaks per minute (SCR) to test whether the VRAPT session could elicit physiological arousal and reactivity. We fitted a generalized linear mixed model with gamma distribution as the data were continuous, positive, positively skewed and over dispersed. In addition, linear mixed effects models were used to examine the effect of time for the early more standardized sessions vs. late personalized sessions on HR, EDA (SCL and SCR), and PR.

## 3 Results

**Figure 5:**
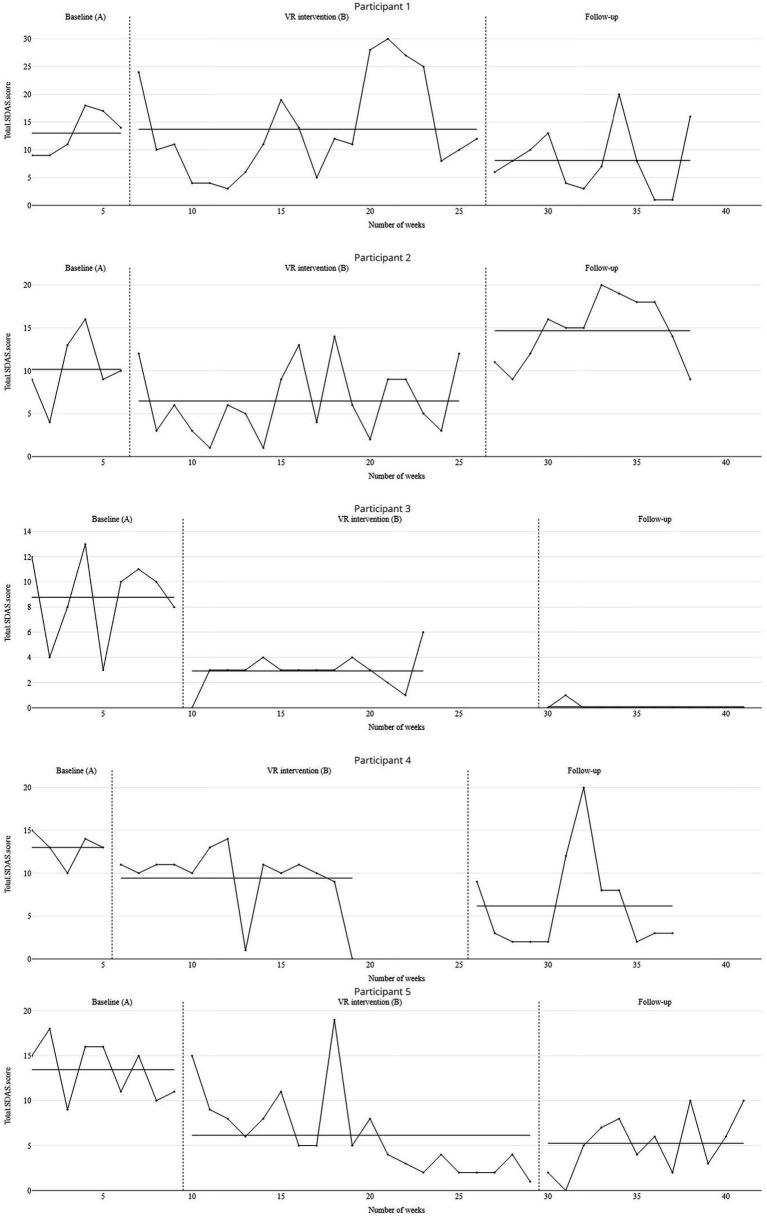
Aggressive behavior over time as measured by the SDAS, visualized per participant. SDAS = Social Dysfunction and Aggression Scale.

Since this was a single case experimental design, analyses are restricted to the completers. The non-concurrent design precludes causal interpretability. Regarding the number of assessment points per participant, Figure 5 shows the number of weeks and sessions. The following results are reported for the five participants who completed the entire intervention and follow-up. These participants are referred to as participants 1 to 5.

### 3.1 Aggressive behavior

**Figure 4:**
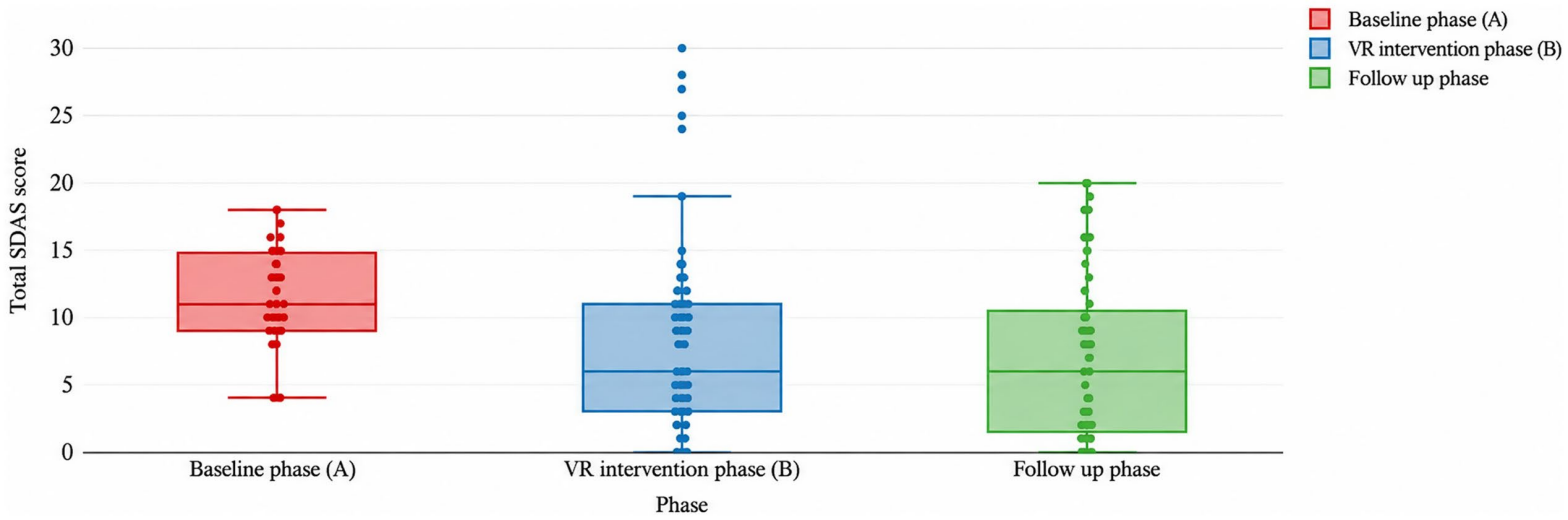
Distribution of scores on the SDAS between the different phases. Boxplot distribution of the total SDAS scores of all participants.

In total, 182 SDAS questionnaires were scored. Of these, 165 were completed by social workers, either digitally via Qualtrics or on paper, and 17 were missing. These 17 missing SDAS questionnaires were completed retrospectively by a single research intern, based on daily reports completed by staff twice daily. These questionnaires were drawn from multiple patients and from different weeks of the study. To minimize potential observer bias, the research intern was blinded to the identity of the participant when completing the scoring instrument. The total mean SDAS score of the five participants who completed the intervention during the baseline phase was *M* = 11.7, *SE* = 0.63 (95% CI [11.12, 12.02]). During the intervention phase, the mean score was *M* = 7.59, *SE* = 0.68 (95% CI [7.31, 8.28]), and during the follow up phase, it was *M* = 6.85, *SE* = 0.81 (95% CI [6.27, 7.43]). The median scores were 11 [9–14.5] in the baseline phase, 6 [3–11], in the intervention phase, and 6 [1.75–10.25] in the follow-up phase. The distribution of the total SDAS scores is shown in Figure 4. Inspection showed there were no missing values. Frequency analysis and descriptive statistics were used to examine the potential presence of abnormal response patterns, and no deviations were found.

Figure 5 shows the time series graphs for individual completing participants under different experimental conditions. The graphs indicate improvements in the total SDAS score when comparing the baseline phase to the intervention phase for participants 2, 3, 4 and 5. Improvements were also observed when comparing the baseline phase to the follow up phase for participants 1, 3, 4 and 5. Visual analysis reveals a decreasing trend in the total SDAS score for participant 1, 3, 4 and 5. Only participant 2 did not show a downward trend, with a higher total SDAS score during the follow up phase (*M* = 14.67) in comparison to the baseline phase (*M* = 10.17).

The stability criterion was largely not satisfied. It was met only for the follow up phase in participant 3 (100%) and for the baseline phase in participant 4 (80%). Overall, 13.33% of the data from all phases fell within a 15% range of the median for each phase. Limited phase stability restricts the interpretability of the results (Table 2).

**Table 2:** Nonoverlap of all pairs (NAP), Tau-U approach and typicality of level change (TLC) estimate.

Participant	Baseline vs. Intervention			Baseline vs. Follow up		
	NAP	Tau-U	TLC estimate	NAP	Tau-U	TLC estimate
1	55.8	−0.117	0.35	79.2*	−0.583*	0.92*
2	73.7*	−0.474	0.94*	21.5	0.570	0.16
3	96.8**	−0.937**	1**	100**	−1**	1**
4	82.1*	−0.642*	0.85*	90*	−0.800*	0.99**
5	90.3*	−0.806**	1**	97.2**	−0.944**	1**

NAP ranges interpreted as weak effects: <65, moderate effects: 66–92; strong effects: ≥93, Tau-U weak effects: 0–0.65, moderate effects: 0.66–0.92; strong effects: ≥0.93 and TLC estimate weak effects: 0–0.65, moderate effects: 0.66–0.95; strong effects: 0.95–1.0. *p < 0.05, **p < 0.001.

Non-overlap of All Pairs (NAP) compares every possible data point from one phase with every data point from another phase and calculates the proportion of pairs where the intervention or follow-up phase data points are better than the baseline data points. NAP analysis between the baseline and intervention phases showed a weak effect for participant 1, moderate effects for participants 2, 4 and 5, and a strong effect for participant 3. Comparing the baseline with the follow-up phase, NAP indicated a weak effect for participant 2, moderate effect for participants 1 and 4, and strong effects for participants 3 and 5. The results are presented in Table 3.

**Table 3:** Effect sizes by Hedges’ g of measured aggressive behavior over time after randomization.

Participant	Baseline vs. Intervention		Baseline vs. Follow up	
	Effect size *g*	*p*-value	Effect size *g*	*p*-value
1	−0.088	0.549	0.918	0.058
2	0.897	0.037	−1.163	0.021
3	2.463	0.001	3.899	0.001
4	0.99	0.06	1.407	0.029
5	1.711	0.001	2.561	0.001
Overall		0.001		0.012

Effect sizes interpreted as 0.2 = small, 0.5 = medium and 0.8 = large.

Unlike NAP, Tau-U accounts for non-overlap between phases, trends during the intervention phase, and can correct for any baseline trends. Tau-U was significant for participant 3 (*τ* = −0.937, *p* < 0.001), participant 4 (*τ* = −0.642, *p* < 0.05), and participant 5 (*τ* = −0.806, *p* < 0.001) when comparing the baseline phase with the intervention phase. When comparing the baseline phase with the follow-up phase, Tau-U was significant for participant 1 (*τ* = −0.583, *p* < 0.05), participant 3 (*τ* = −1, *p* < 0.001), participant 4 (*τ* = −0.800, *p* < 0.05) and participant 5 (*τ* = −0.944, *p* < 0.001). The results are presented in Table 3.

Unlike NAP and Tau-U, TLC does not face issues of typicality. TLC analysis showed a weak effect for participant 1, a moderate effect for participant 4, and strong effects for participants 2, 3 and 5 when comparing the baseline with the intervention phase. When comparing the baseline with the follow-up phase, TLC showed a weak effect for participant 2, a moderate effect for participant 1, and strong effects for participants 3, 4 and 5. The results are presented in Table 3.

Effect sizes were calculated using Hedges’ *g* to measure changes in aggressive behavior over time after randomization. When comparing the baseline phase to the intervention phase, participants 2, 3, 4, and 5 showed large effect sizes, indicating a significant reduction in aggression. Similarly, when comparing the baseline phase to the follow-up phase, participants 2, 3, 4, and 5 showed large effect sizes, though participant 2 showed a large effect size in the opposite direction. See Table 3 for the results.

### 3.2 Arousal

**Figure 6:**
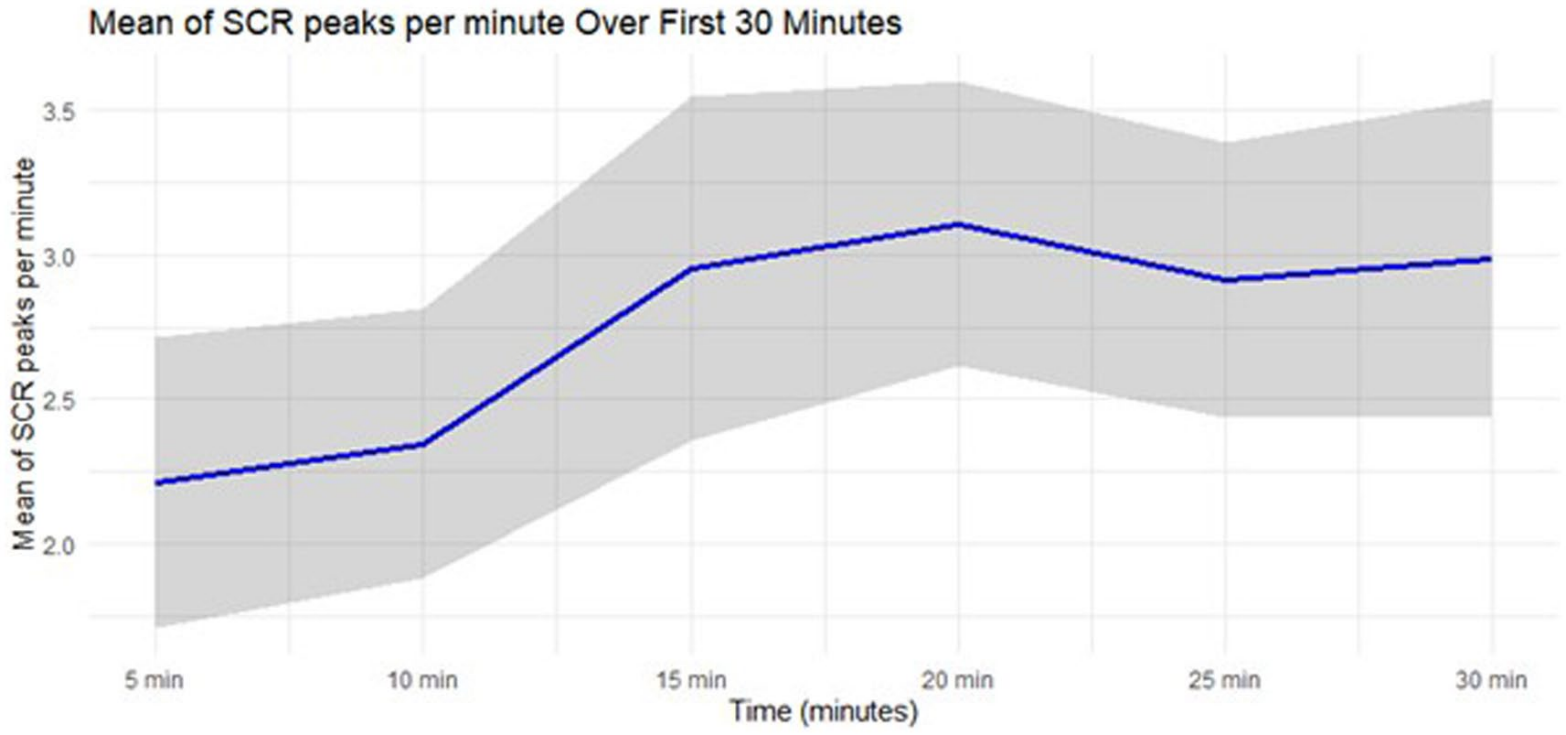
Mean of electrodermal activity (EDA) over the first 30 min during the session.

PR, PRV and EDA (SCL and SCR) were analyzed. For the first 30 min of each session. Although data beyond 30 min were considered, some sessions lasted only 30 min; therefore, analyses were restricted to the initial 30 min. These 30-min intervals were divided into six 5 min-epochs (de Looff et al., 2019). The analysis resulted in a significant effect of time on SCR peaks per minute (*b* = 0.077, *SE* = 0.039, *z* = 1.97, *p* = 0.049), which translates to an approximate 8% increase in SCR peaks per additional 5-min interval. As expected, the increase in SCR indicates a rise in arousal during the first 30 min of the sessions, averaged across all sessions. This is shown in Figure 6. Consistent with our hypothesis, an increase in arousal (sympathetic activation) was observed during a VR session. No effect of time on the SCL was observed.

The analysis showed no effect of time for early standardized (session 1 through 6) versus late personalized sessions (session 7 through 12) indicating no differences in sympathetic arousal between conditions. Contrary to the hypothesis, the more personalized sessions did not elicit higher arousal. All analyses controlled for temperature, accelerometry and session, but did not significantly improve model fit nor explain additional variance. Contrary to expectations, there was no difference between the first six sessions and the last in terms of arousal.

No increase in PR overtime was observed. Regarding pulse rate variability (PRV), measured using RMSSD, a substantial amount of data was missing probably due to extensive physical movement with the wristband, which introduced numerous artifacts. As a result, the PRV measure was deemed unsuitable for analysis. Analyses of physiological responses indicated increases in SCR during the VR role-play sessions, suggesting elevated physiological arousal. However, other physiological indicators did not show consistent patterns, and PRV data were largely unusable due to data loss and signal artifacts. Therefore, these physiological findings should be interpreted with caution and regarded as exploratory.

### 3.3 Therapeutic alliance

By analyzing the SRS, the influence of VRAPT-ID on therapy therapeutic alliance was examined. The results for the five participants who completed the VRAPT-ID intervention are presented in Supplementary Table 1. All mean scores of the participants are rated positively across the four SRS elements, including Listening (*M* = 87.402), Importance (*M* = 87.525), Content (*M* = 85.147), and Total (*M* = 85.405). Additionally, all scores had a high median, ranging from 80 to 92. Therefore, all four elements enhanced therapeutic alliance.

T1 consisted of SRS data from sessions 1 to 5, while T2 consisted of data from sessions 6 to 12, which included interactive role-plays based on participants’ own experiences. Comparing T1 to T2, participant 2 showed a strong improvement in Listening (NAP score > 93). Regarding Importance, three participants showed medium effects of improvement (NAP > 66). For Content, two participants showed medium effects of improvement over time. In the Total score, one participant showed a strong effect, and another showed a medium effect over time. All NAP scores are presented in Supplementary Table 2.

Supplementary Table 3 presents the qualitative evaluation of the five completing participants. The five topics evaluated were (1) liking to participate in VR treatment, (2) connection with the trainer, (3) helping to deal with anger, (4) continuing with VRAPT-ID, and (5) continuing VR in general. Participants responded predominantly positively. All five completing participants agreed that VRAPT-ID helped them better manage their anger and found it enjoyable. Three out of the five completing participants specifically mentioned SODA as helpful in dealing with their anger. All five completing participants were positive about their connection with their VR trainer, appreciating the time taken to explain and make them feel at ease. Except for participant 3, the other four participants expressed a desire to continue with VRAPT-ID and VR training in general. Suggestions were VR training for addiction and VR for crossing own boundaries to belong. They suggested using VR training for substance addiction and for learning to monitor their own boundaries.

## 4 Discussion

The aim of this pilot study was to conduct a feasibility analysis of the effect of VRAPT-ID on aggressive behavior in individuals with MBID convicted of a violent crime residing in an FPC under a TBS order. We found preliminary indications that VRAPT-ID may decrease aggressive behavior. The results show a decrease in four out of five completing participants. In addition, we hypothesized that arousal levels would increase during the VR sessions, which was evident in SCR, but not PR and PRV Arousal levels increased during VR training in three participants, suggesting an adequate trigger. Lastly, the hypothesis that VRAPT-ID enhances therapeutic alliance was confirmed. All five completing participants agreed VRAPT-ID helped them manage their anger more effectively and found VRAPT-ID enjoyable. All elements of the SRS reflected a strengthened therapeutic alliance. These findings offer preliminary indications for potential relevance of VRAPT-ID in reducing aggressive behavior in individuals with MBID in forensic settings. However, the results should be interpreted with considerable caution given the pilot nature of the study, the heterogeneity within individuals with MBID and the limitations of the applied multiple baseline single case experimental design, as further described in section 4.2.

Results of several recent studies on VRAPT derivatives are in line with our findings. As mentioned earlier, Woicik et al. (2023) reported positive findings on aggression, anger, and emotion regulation in a pilot study in a prison-based population (N = 17). A limitation mentioned by Woicik et al. (2023) was that the inclusion of participants was based on self-referral, which may have led to selection bias, and the lack of documentation on reasons for participation. In the current study, participants were referred by their head of treatment, with documented reasons for refusal and discontinuation. Another limitation of the study of Woicik et al. (2023) was the reliance on self-report, which we avoided for the primary outcome of aggressive behavior.

When examining arousal levels, skin conductance responses (electrodermal activity; EDA) increased during VR sessions, suggesting activation of the sympathetic nervous system. Elevated arousal might also be the result of increased physical activity during the VR sessions, although we controlled for movement and the results suggested that the sympathetic activation was not solely driven by physical activity. Elevated arousal during VR training is assumed to reflect adequate elicitation of physiological reactivity with the VR stimuli. This is important as sympathetic activation is expected to facilitate learning processes, consistent with the General Aggression Model and Bandura’s social learning theory (Allen et al., 2018; Bandura, 1978; Roozendaal et al., 2009). Increased arousal alone is, however, not sufficient for optimal experiential learning. The Yerkes-Dodson law of optimal arousal suggests that excessive arousal might decrease learning, and we were unable to determine whether participants were in the zone of optimal arousal in the current study (Broadhurst, 1957; Yerkes and Dodson, 1908). Consequently, although increased arousal may enhance learning performance, it is essential to ensure that arousal levels do not become excessively elevated during VR sessions. Although an overactivated stress response was not expected because of VR intervention, future research should account for individual differences in stress system activity, such as those related to trauma history. Additionally, some participants – particularly those with social anxiety – may experience excessive arousal during VR exposure because of the provoking social situations.

Arousal was assessed using EDA (SCL and SCR), pulse rate variability (PRV), and pulse rate (PR). No increase in PR was found during the VR sessions, which may be explained by the fact that PR, although expected to increase with higher arousal, reflects the combined activity of both autonomic branches while SCR is a measure of sympathetic activity. Due to a substantial amount of missing PRV data, PRV could not be included in the analysis. Therefore, the physiological findings should be interpreted with caution. Although increases in the SCR component of EDA suggest elevated physiological arousal during the VR role-play sessions, PR did not show consistent patterns and PRV were largely unusable due to data loss and signal artifacts. Consequently, these findings cannot be interpreted as evidence of a confirmed physiological mechanism underlying the intervention effects. Instead, they should be considered exploratory and warrant further investigation in future studies using more robust physiological measurement procedures.

Wrist-worn devices are known to suffer from loose connections, poor electrode placement or movement artifacts, among other elements, and may not have been optimal for capturing physiological signals during VR sessions (Milstein and Gordon, 2020; Schuurmans et al., 2020). Future studies should consider using more accurate devices, such as chest straps or patches which are better suited to conditions involving significant physical movement. Finally, future research could improve the interpretation of physiological data by differentiating biomarker measurements across specific time points within sessions, for instance distinguishing between different phases or types of role-playing exercises. Differentiating physiological responses across these distinct moments would allow opportunities to capture more fine-grained fluctuations in arousal and autonomic regulation.

Regarding therapeutic alliance, both SRS data and qualitative questionnaires revealed a positive attitude towards VRAPT-ID, despite the high dropout rate. Of the 19 patients enrolled, only 10 started, with dropouts primarily due to the absence of a reward and concurrent studies. External rewards stimulate participation, especially for patients with MBID (Constantin et al., 2017). Insufficient consideration of other studies in the same clinic led some participants to choose another study involving VR that offered a reward, even though participation in both studies was allowed. Five of the 10 participants dropped out of VRAPT-ID. This high dropout rate might suggest that many patients did not find the training enjoyable enough to continue. However, a closer examination reveals that all five participants who dropped out did so after only one to three sessions, and the reasons were primarily external and not related to the intervention. The VR trainers indicated that starting role-plays earlier, ideally in sessions 1 or 2, might have retained participation. Initial sessions primarily involved orientation in the VR environment, with role-plays starting around sessions 4 or 5. Some patients felt the progression and pace were too slow. This highlights the importance of personalizing the session structure, as suggested by Geraets et al. (2023). A patient who has been in the clinic for years likely already possesses emotional discernment and could start role-plays sooner. The gamification element of VR training has the potential to support the growth of motivation (Xu et al., 2021). Moreover, high levels of dropout rates are common in forensic psychiatric populations (Bruinsma et al., 2020). Regarding clinical implications, the use of VR in therapy aligns with the Risk-Need-Responsivity (RNR) model (Bonta and Andrews, 2007). With respect to the Need principle, virtual reality is applicable in forensic psychiatry, as a significant challenge in treatment is the limited experience and exposure to diverse real-world scenarios resulting from the high-security environment. The Responsivity principle is especially important in the heterogeneous group of patients with MBID whose treatments should be adapted (Didden et al., 2019; Janković et al., 2021), ultimately aiming to reduce recidivism (Nouwens et al., 2017; Polaschek, 2012).

### 4.1 Strengths

A strength of the current pilot study is the use of personalized role-plays in a simulated VR environment to treat aggression regulation problems. This approach allows patients to immerse themselves in realistic environments comparable to the outside world, where they can practice behavior and overcome difficulties (Emmelkamp and Meyerbröker, 2021). VR role-plays provide patients with MBID with deeper insights into interactions by emotionally experiencing interpersonal conflicts (Holsteg et al., 2024). Another strength is that they can practice in a safe environment with little risk of harm or injury. Another strength of this study was the high motivation of all trainers, resulting in an enthusiastic and eager-to-learn group of 11 trainers. While this large number may impact reliability due to individual therapeutic styles and varying skills in performing and improvising role-plays, it also reflects clinical practice more accurately. It is impractical for just one or two trainers to conduct all VR sessions, and the larger group reduces vulnerability if trainers drop out or leave. Lastly, the involvement of the participants’ social network was considered a benefit, specifically social workers from the ward who participated in two sessions. As suggested by Geraets et al. (2023), involving the network encouraged participants to complete the training and led to greater generalization of learned behavior on the ward. Additionally, social workers provided valuable input of patients’ behavior and aggressive triggers, helping the development of relevant VR scenarios. This involvement facilitated a two-way exchange of information.

### 4.2 Limitations

Various limitations should be considered when interpreting our results. First, based on this pilot study, VRAPT-ID appears to be associated with reductions in aggressive behavior. However, the SCED design requires careful interpretation because of its small sample size and absence of a comparison group. Additionally, restricting analyses to study completers introduces selection bias, as the restriction to completers (*n* = 5) in the presence of substantial dropout further limits interpretability and increases the risk that results reflect a selective subgroup of more motivated or responsive participants. Second, limited phase stability and the non-concurrent design preclude causal interpretability of the results. Additionally, the non-concurrent design may be sensitive to confounding in an inpatient forensic setting, where shared environmental factors (e.g., staff changes or ward climate) may have influenced outcomes independent of the intervention. Third, the comprehensiveness of the measurement instruments for assessing aggressive behavior poses a problem. The GAM focuses on various components of aggression and this study solely focused on observed aggressive behavior. We only rated observed aggressive behavior without self-report. Operationalizing aggressive behavior and social skills in questionnaires and observation lists remains difficult. Fourth, the missing SDAS questionnaires were scored retrospectively by a single research intern, which may have had implications for measurement consistency and observer bias. Regarding treatment integrity, no formal fidelity assessment was conducted. The therapeutic alliance scores were relatively high, suggesting a potential ceiling effect that may have limited variability in the measure. Lastly, a significant number of artifacts were present in the physiological data collected with the Empatica E4. As a result, only EDA and PR could be used to investigate arousal patterns during sessions. Our study does not provide a direct test of the hypothesized mechanism linking arousal to behavioral change, and the physiological findings were limited in consistency and completeness.

### 4.3 Future research

As this was a pilot study, the next step is that we will test VRAPT-ID in an RCT to validate the intervention, as suggested in recent VR research (Geraets et al., 2023; Ivarsson et al., 2023; Woicik et al., 2023). This is necessary to obtain robust evidence of effectiveness for this aggression regulation training and allow for generalization. Regarding future research, the strength of VRAPT-ID lies in its use of personalized role-playing scenarios based on the patient’s life. However, this personalization results in a lack of standardization, posing a challenge for scientific substantiation of effectiveness. Future studies should balance personalized input with greater standardization to better elucidate the effective elements of the training. To address the high dropout rate, future research should include broader support network, such as social workers during training. This is crucial given the importance of interpersonal context for the MBID population (Surley and Dagnan, 2019). In the forensic context, this means engaging social workers and important relatives that are in contact with the patient. During this study, network members participated in only two sessions. A follow-up study should actively involve the social network, specifically social workers, from the outset and include them in multiple sessions. To encourage participants to complete the training, it is necessary to personalize the pace of the VR sessions. As Willner (2006) stated, personalizing and simplifying therapy can increase readiness for therapy in people with MBID, which might be necessary for some individuals with MBID to engage in VR training. Regarding the optimal regulatory window, understanding an individual’s baseline arousal is essential to ensure that arousal remains within an optimal range throughout the session. Establishing this homeostatic range might be an interesting avenue for future research. In addition, the heterogeneity within individuals with MBID should be considered by examining relevant covariates. Variability in cognitive functioning, adaptive skills, and co-occurring difficulties may moderate treatment processes and outcomes. Finally, future research should more directly examine the proposed mechanism underlying VRAPT-ID. For instance, within-person or time-lagged analyses could assess whether changes in arousal are associated with subsequent changes in aggressive behavior. Also, using a multimethod approach to assess arousal (e.g., physiological, observational, and self-report measures) and including intermediate process measures such as emotion regulation or skill acquisition may provide a more robust test of the hypothesized mechanism.

## 5 Conclusion

The results of this pilot study suggest that VRAPT-ID may be a feasible and potentially promising intervention for addressing aggressive behavior in individuals with MBID in a forensic setting, supporting previous research (Geraets et al., 2023; Woicik et al., 2023). Among the participants who completed the intervention, reductions in aggressive behavior were observed in four out of five individuals. Although the findings are preliminary, they may indicate that applying VRAPT-ID could be associated with increased physiological arousal during training sessions. However, the current study does not provide a direct test of the proposed mechanism linking arousal to behavioral change. The study also underscores the importance of personalized role-plays and the involvement of the social network in supporting the intervention. Future research, including RCT, is needed to further examine these preliminary findings and to investigate the proposed underlying mechanisms of VRAPT-ID. The findings must be interpreted as preliminary indications of feasibility rather than evidence of effectiveness.

## Data Availability

The raw data supporting the conclusions of this article will be made available by the authors, without undue reservation.

## Footnotes

1
http://www.randomizer.org
