# Supplementary material for: Reducing aggressive behavior in forensic inpatients with virtual reality aggression prevention training—intellectual disability: a pilot study
Source: Front Psychol. 2026 May 29;17:1792178. doi: 10.3389/fpsyg.2026.1792178 (PMC13259759; doi:10.3389/fpsyg.2026.1792178)
Supplement: Supplementary file 1 [file Table_1.docx]

Supplementary Material

# Supplementary Tables

**Supplementary Table 1**

*SRS Mean score*

| Question | Overall Mean | Participant | Mean | Median |
| --- | --- | --- | --- | --- |
| 1 Listening | 87.402 | 1 | 79.500 | 80.00 |
|  |  | 2 | 86.583 | 88.00 |
|  |  | 3 | 92.000 | 92.00 |
|  |  | 4 | 88.429 | 88.50 |
|  |  | 5 | 90.500 | 90.50 |
| 2 Importance | 87.525 | 1 | 86.349 | 80.0 |
|  |  | 2 | 84.750 | 89.00 |
|  |  | 3 | 92.000 | 92.00 |
|  |  | 4 | 86.357 | 87.50 |
|  |  | 5 | 88.167 | 90.50 |
| 3 Content | 85.147 | 1 | 77.000 | 80.00 |
|  |  | 2 | 82.750 | 85.00 |
|  |  | 3 | 91.833 | 92.00 |
|  |  | 4 | 87.071 | 88.00 |
|  |  | 5 | 87.083 | 90.00 |
| 4 Total | 85.405 | 1 | 77.500 | 79.50 |
|  |  | 2 | 83.667 | 86.00 |
|  |  | 3 | 91.667 | 92.00 |
|  |  | 4 | 83.357 | 86.50 |
|  |  | 5 | 90.833 | 91.00 |

*Note.* Max. score *= 92.*

**Supplementary Table 2**

*NAP T1 – T2*

| Question | Participant | No. Positive Pairs | No. Negative Pairs | No. Ties | Total no. Pairs | NAP | CI |
| --- | --- | --- | --- | --- | --- | --- | --- |
| 1 Listening | 1 | 14 | 14 | 7 | 35 | 50.0 | [0.16–0.84] |
|  | 2 | 34 | 1 | 0 | 35 | **97.1** | [0.83–1.00] |
|  | 3 | 18 | 0 | 25 | 35 | 33.3 | [0.50–0.50] |
|  | 4 | 10 | 10 | 10 | 45 | 20.0 | [0.09–0.67] |
|  | 5 | 2 | 0 | 35 | 50 | 33.3 | [0.00–0.47] |
| 2 Importance | 1 | 20 | 11 | 4 | 35 | 62.9 | [0.23–0.91] |
|  | 2 | 22 | 9 | 4 | 35 | *68.6* | [0.30–0.94] |
|  | 3 | 0 | 0 | 35 | 35 | 50.0 | [0.50–0.50] |
|  | 4 | 14 | 26 | 8 | 48 | 37.5 | [0.06–0.70] |
|  | 5 | 27 | 2 | 6 | 35 | *85.7* | [0.61–1.00] |
| 3 Content | 1 | 20 | 9 | 6 | 35 | 65.7 | [0.36–0.93] |
|  | 2 | 31 | 4 | 0 | 35 | *88.6* | [0.66–1.00] |
|  | 3 | 7 | 0 | 28 | 35 | 60.0 | [0.50–0.80] |
|  | 4 | 13 | 32 | 3 | 48 | 30.2 | [0.00–0.62] |
|  | 5 | 27 | 3 | 5 | 35 | *84.3* | [0.54–1.00] |
| 4 Total | 1 | 13 | 15 | 7 | 35 | 47.1 | [0.11–0.80] |
|  | 2 | 35 | 0 | 0 | 35 | **100.0** | [1.00–1.00] |
|  | 3 | 21 | 0 | 14 | 35 | *80.0* | [0.60–1.00] |
|  | 4 | 11 | 31 | 6 | 48 | 29.2 | [0.00–0.65] |
|  | 5 | 16 | 10 | 9 | 35 | 58.6 | [0.20–0.93] |

*Note.* No formatting = 0-65% weak effect; *italic* = 66-92% medium effect; **bold** = 93-100% strong effect.

**Supplementary Table 3**

*Qualitative evaluation of the completing participants*

| Question | Participant | Response |
| --- | --- | --- |
| 1. How did you feel about participating in this VR treatment? What did you like/dislike about it? | Participant 1 | Nice to participate. That it also helps. And another way to learn something. |
|  | Participant 2 | Fun and educational and I have become aware of my learning goals. |
|  | Participant 3 | I like the therapy, recognizing emotions and applying SODA. |
|  | Participant 4 | Fun: educational; how to deal with anger. Not nice: space too small. |
|  | Participant 5 | I learned a lot from it. I have learned to deal with anger using SODA. |
| 3. Has VRAPT-ID helped you deal with your anger better? | Participant 1 | Yes, I haven't used it yet. |
|  | Participant 2 | Yes, I now know where my limit is. |
|  | Participant 3 | Yes. |
|  | Participant 4 | Yes, I learned to apply SODA and I can name all the steps. Staff notices that I apply it. |
|  | Participant 5 | Yes, I now use SODA. SODA makes me think and act accordingly. |
| 4. Do you want to continue with VRAPT-ID treatment? | Participant 1 | Yes. |
|  | Participant 2 | Yes. |
|  | Participant 3 | No, I've learned enough. |
|  | Participant 4 | Yes, I find it exciting and educational. |
|  | Participant 5 | Yes. |
| 5. Would you also like to receive VR treatment for other problems in the future? | Participant 1 | Yes. |
|  | Participant 2 | Yes. |
|  | Participant 3 | No. |
|  | Participant 4 | Yes. For example for addiction. |
|  | Participant 5 | Yes, for the fact that I always want to belong and therefore often cross my boundaries. |
